# Orbital fractures and concurrent ocular injury in a New Zealand tertiary centre

**DOI:** 10.3389/fopht.2023.1305528

**Published:** 2023-11-21

**Authors:** Nicholas J. Theis, Pritesh Narsinh, Samuel Newlands, Jason Erasmus, Rebecca Stack

**Affiliations:** ^1^ Department of Ophthalmology, Te Whatu Ora Waitaha Canterbury, Christchurch, New Zealand; ^2^ Department of Oral and Maxillofacial Surgery, Te Whatu Ora Waitaha Canterbury, Christchurch, New Zealand

**Keywords:** facial trauma, ocular injury, orbit, orbital fractures, ophthalmology

## Abstract

**Background:**

Orbital fractures are a common presentation to acute care and carry an associated risk of ocular injury, however, previous research has not investigated injury rates by fracture category. These patients are frequently assessed by non-ophthalmic clinicians, however, limited data exists regarding referral patterns and how this impacts recorded injury rates (1–3).

**Methods:**

We performed a retrospective review of all orbital fractures presenting to a tertiary hospital in Christchurch, New Zealand between March 2019 and March 2021. Data including mechanism of injury, fracture type, demographic characteristics, and associated ocular injury were recorded.

**Results:**

284 patients with orbital fractures were identified. 41% of patients had isolated wall fractures, while 59% had complex orbitofacial fractures. Fractures were more common in males, and occurred more frequently in young individuals. The most common mechanism of injury was interpersonal violence (32%), followed by falls (23%). 41% of patients were reviewed by ophthalmology (*n* = 118). Of those, 33% had an associated ocular injury. Severe ocular injury (defined as vision threatening, requiring globe surgery or acute lateral canthotomy and cantholysis) occurred in 4.9% of those with formal ophthalmic review. 0.7% of patients required intraocular surgery or lateral canthotomy due to their orbital fracture.

**Conclusion:**

Orbital fractures have a high rate of concurrent ocular injury in our study population, though rates of subsequent intraocular surgery are low. There was no significant difference in injury rates between isolated and complex fracture categories. Vision-threatening ocular injury occurred in 4.9% of fractures.

## 1 Introduction

Maxillofacial trauma is a common reason for presentation to acute care, with potentially devastating functional and cosmetic consequences. Approximately one-fifth of facial fractures occurring in New Zealand involve the orbital bones (1). The most common mechanisms of injury internationally are motor vehicle accidents and interpersonal violence, with males more commonly affected than females and with most fractures occurring in patients under the age of 30 (2, 3).

Orbital fractures carry a risk of associated ocular and periocular injury by nature of their anatomical proximity to the globe, extraocular muscles, and orbital connective tissues. Isolated fractures involving a single wall appear to carry a lower risk than complex orbitofacial fractures (4, 5). Rates of ocular injury based on the international literature range from 2.7-13.7%, and this variability in reported rates may relate to inconsistent classification of ocular injury across studies. Presenting features associated with a higher risk of concurrent ocular injury based on the existing literature include reduced visual acuity (VA), presence of an afferent pupillary defect, and restricted motility (6).

Research to date has focused predominantly on demographics, mechanisms of injury, and fracture patterns (1, 7, 8). Despite globe injury being a common clinical concern, existing data assessing rates of associated ocular injury is limited. Furthermore, there is limited information regarding rates of referral to ophthalmic specialists from initial acute care assessment, and how long this process takes. This study aimed to describe patterns of orbital fractures in a tertiary New Zealand hospital, along with their associated rate of ocular injury, referral pathways and lead-times to ophthalmic review.

## 2 Materials and methods

We undertook a retrospective review by three investigators of all consecutive orbital fractures referred to the Maxillofacial department at the Christchurch Hospital between the 1^st^ March 2019 to the 31^st^ March 2021. Relevant demographic information including age, sex, ethnicity (using prioritized output), the mechanism of injury, along with the type of fracture sustained and the presence of associated ocular injury, were recorded. Data regarding concurrent ocular injury, time to review by ophthalmology, and features of the presenting ocular examination were also recorded. Fracture characteristics were obtained from written radiology reports, as well as a review of selected radiographic images.

Investigators first classified ocular injuries as present or absent. If an ocular injury was present, it was classified as mild if no treatment was required and no permanent ocular sequelae were identified (e.g. subconjunctival hemorrhage, periocular hematoma, or swelling), moderate if medical treatment was initiated or ophthalmic follow-up was warranted, but no permanent ocular sequelae were identified (e.g. hyphema without raised intraocular pressure, corneal abrasion), and severe if there was a vision threatening injury or permanent vision loss occurred, or if surgical management was required (e.g. traumatic optic neuropathy, orbital compartment syndrome, globe rupture). A normal ophthalmic examination was defined as normal or symmetrical VA, normal pupil responses with no relative afferent pupillary defect (RAPD), and a full range of eye movements (or baseline ocular motility for the patient).

Diplopia, restricted motility, and altered globe position were recorded separately from globe injuries such as hyphema and subconjunctival hemorrhage in order to delineate fracture-related orbital injury from globe injury. Lid-involving facial lacerations and contusions, as well as other facial injuries not involving the orbit or globe, were not included in our analysis.

Complex fractures were categorized into orbitozyomatic, Le Fort, and naso-orbito-ethmoid (NOE) fracture types. While floor and maxillary wall involving fractures are not a commonly documented complex fracture subtype in the existing literature, we included these fractures as a subcategory, as these fractures appeared commonly in our data. Other complex fractures included all complex fractures that did not fall within the aforementioned categories.

Incidence rates were compared using Fischer analysis with rate ratios with mid-p exact 95% confidence intervals calculated. Analysis was conducted with Microsoft Excel and the OpenEpi package 10. All analysis was performed with 2 sided statistical tests considered significant at the 5% level. All data was de-identified prior to analysis.

## 3 Results

**Figure 1:**
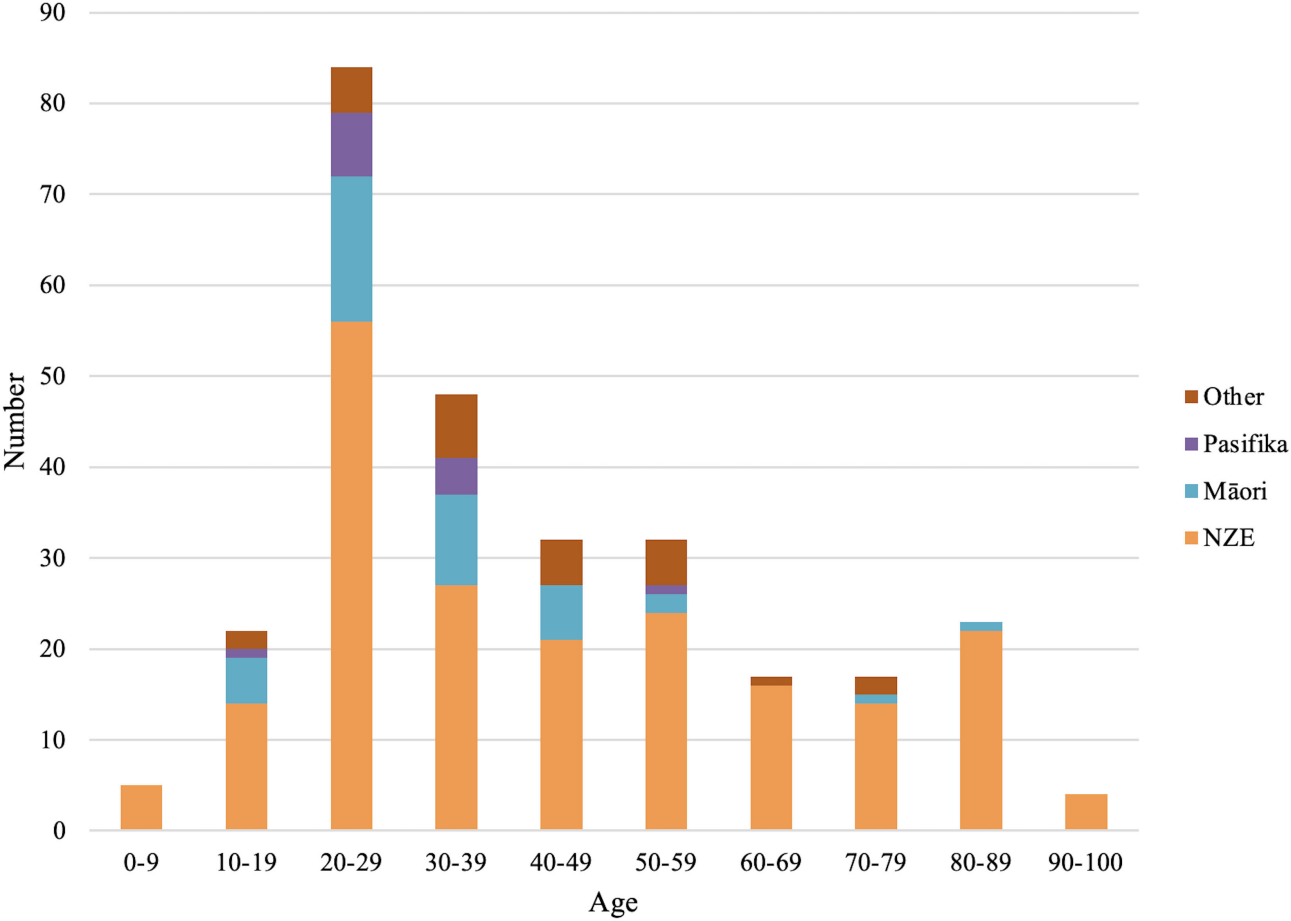
Age of study participants by ethnicity.

284 patients were included for analysis for the timeframe spanning from March 2019 to March 2021. Fractures were more common in males than females, and most occurred in young individuals with a mean age of 42 and a median age of 35 (3-98) years (**Figure 1**). The most common mechanism of injury was interpersonal violence. E-scooter related injuries made up 5% of all fractures. Alcohol intoxication was self-reported in 21% of patients with a documented orbital fracture, while other drug intoxication was reported in 2% (**Table 1**).

**Table 1:** Baseline demographics.

	Number (*n*)	Percentage (%)
Age (years)		
0-9	5	2
10-19	22	8
20-29	84	30
30-39	48	17
40-49	32	11
50-59	32	11
60-69	17	6
70-79	17	6
80-89	23	8
>90	4	1
Gender		
Male	214	76
Female	69	24
Ethnicity		
NZ European	203	72
Māori	41	14
Pacific Island	13	4
Asian	11	4
Other	17	6
Mechanism of injury		
Interpersonal violence	91	32
Sporting injury	50	18
Fall	65	23
Motor vehicle accident	11	4
Bicycle	20	7
E-scooter	15	5
Other	32	11

**Figure 2:**
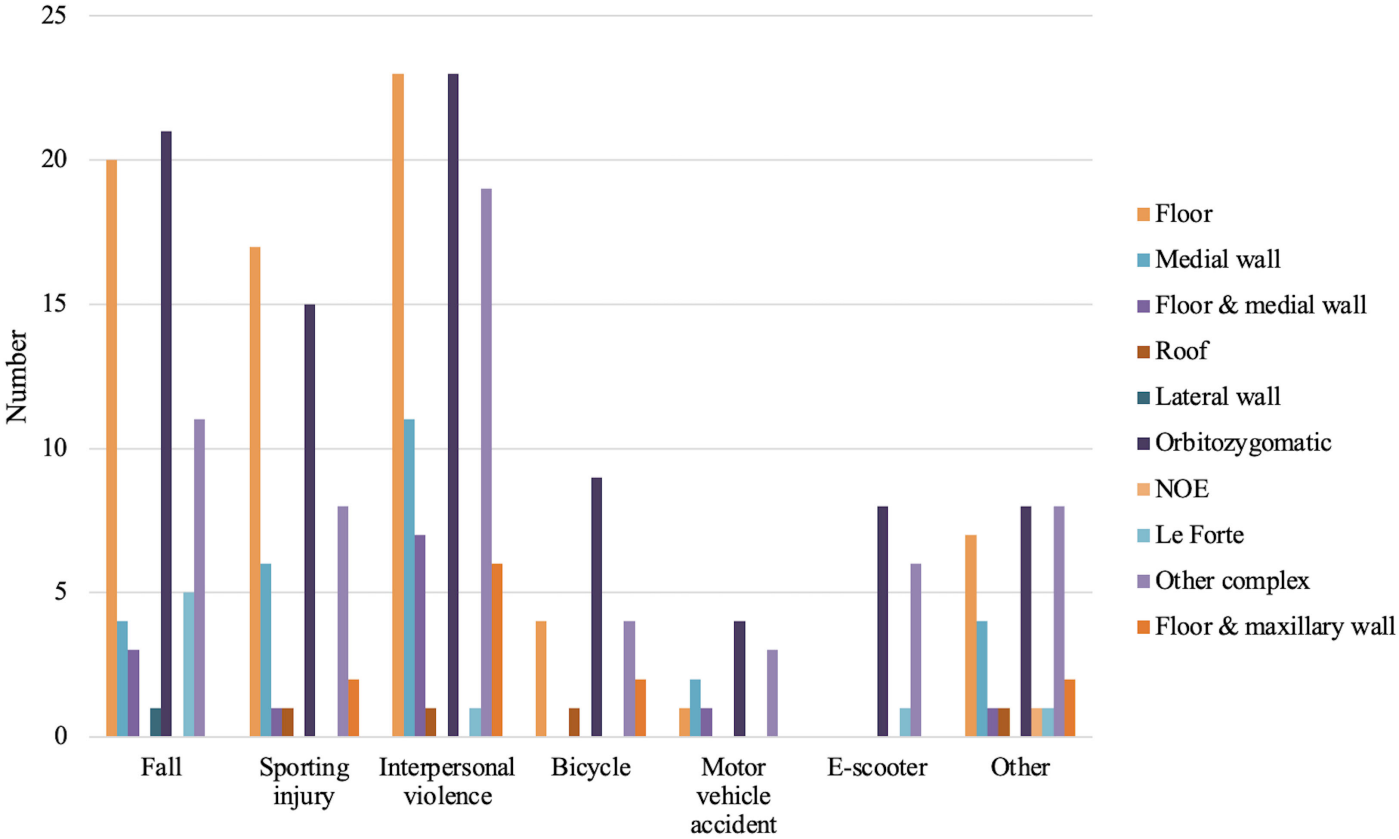
Complex and isolated orbital fractures by mechanism.

Of the fractures identified, 41% were isolated orbital wall fractures involving the roof, floor, medial or lateral walls. Of isolated fracture subtypes, orbital floor fractures were the most common. Complex orbitofacial fractures comprised 59% of fractures, with orbitozygomatic fractures making up the majority. Of the isolated orbital fractures identified, 55% were left-sided, 44% were right-sided, and 1% were bilateral (**Table 2**). Interpersonal violence was the most common mechanism across all fracture categories except for Le Forte fractures, which occurred more frequently due to falls (**Figure 2**).

**Table 2:** Orbital fractures by anatomical subtype.

Fracture Type		Number (*n*)	Percentage (%)
*Isolated*	Floor	72	62
	Medial wall	27	23
	Floor & medial wall	13	11
	Roof	3	3
	Lateral wall	1	1
*Complex*	Orbitozygomatic	88	52
	NOE	1	1
	Le Forte (I & II)	8	5
	Floor & maxillary wall	12	7
	Other complex	59	35
*Total*	Isolated	116	41
	Complex	168	59

**Figure 3:**
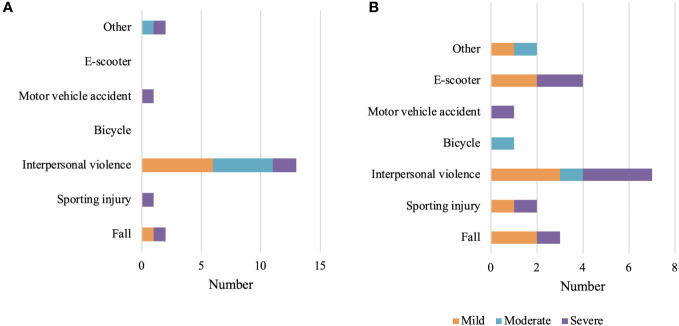
Ocular injuries by mechanism. **(A)** Isolated fractures, **(B)** Complex fractures.

A total of 118 patients were reviewed by ophthalmology. The mean time to formal ophthalmology review was 13.2 days (range 0-116 days). Of those reviewed by the ophthalmology department, 33% had an associated ocular injury. Severe ocular injury occurred in 12% of these patients and was more common in complex fractures than in isolated fractures (**Table 3**). Interpersonal violence was the most common mechanism associated with ocular injury across all fracture types (**Figure 3**). 0.7% of patients required intraocular surgery as a result of their orbital fracture (**Table 4**).

**Table 3:** Rates of ophthalmic review and ocular injury by fracture category.

	Isolated		Complex		Total		
	n	%	n	%	*p*-value*	n	%
*Total Patients*	116		168			284	100
*Normal exam other specialty*	46	40	61	36	0.88	107	38
*No record of any eye exam*	11	9	48	29	<0.05	59	21
*Total reviewed by ophthalmology*	59	51	59	35	<0.05	118	42
No injury**	40	68	39	66		79	33
Any injury**	19	32	20	34		39	33
Mild	7	12	9	15	0.80	16	14
Moderate	6	10	3	5	0.51	9	8
Severe	6	10	8	14	0.79	14	12

*p-values calculated using two-tailed tests of statistical significance.

**in those patients formally reviewed by ophthalmology.

**Table 4:** Severe ocular injuries by fracture category.

Injury type	Isolated	Complex	Total
	(n = 6)	(n = 8)	(n = 14)
Hyphaema with raised IOP	2	3	5
Retrobulbar haemorrhage	1	2	3
Penetrating eye injury	1^†*^	0	1
Retinal detachment	1^†^	0	1
Choroidal rupture	1	0	1
Traumatic optic neuropathy	0	3	3

*penetrating eye injury due to a fall onto sharp object, with concurrent orbital fracture.

†required intraocular surgery.

Of patients who were not formally reviewed by ophthalmology, 65% had a documented ophthalmic examination by another specialty service with no significant ocular injury identified (either Emergency Medicine or Maxillofacial Surgery). There was no formally documented ophthalmic exam in 29% of complex fractures and 9% of isolated fractures (**Table 3**). The baseline characteristics of patients who were not formally reviewed by ophthalmology were comparable to those in patients who did have a formal ophthalmic examination by an ophthalmologist (**Appendix 1**). Patients reviewed by ophthalmology were, on average 6 years younger than those who were not referred for ophthalmic review, and this was statistically significant (*p* = 0.02).

## 4 Discussion

This study captured 284 patients presenting over a 2 year period to a tertiary hospital with one or more diagnosed orbital or orbit-involving facial fractures. Fractures occurred predominantly in young, male individuals, and interpersonal violence was the most common cause. This is consistent with previously reported demographics within New Zealand and internationally (1, 2, 7).

We observed high ocular injury rates associated with these fractures, however, overall identified injury rates were not statistically different between isolated and complex fracture categories. Complex fractures were less likely to have a documented eye exam and ophthalmology review occurred less in complex fractures compared with isolated fractures, however, the identified rates of ocular injury were similar between the two groups. It is plausible that fewer complex fractures were reviewed by ophthalmology as there were more significant concurrent injuries at the time of presentation in these fractures, which may distract the evaluating practitioner from ocular assessment. Some patients with complex fractures were unable to be assessed at initial presentation due to being critically unwell, and this may also have contributed to the resultant lower rate of ophthalmic review in this population. Overall, injury rates in our study are high compared with international rates, with an ocular injury identified in a third of orbital fracture patients reviewed by our ophthalmology service compared with estimates ranging from 2.7%-13.7% globally (6). Interpersonal violence was the most common fracture mechanism associated with ocular injury in both isolated and complex fracture categories (**Figure 3**), which is consistent with the existing literature (5, 9).

Less than 50% of all orbital fractures were referred to ophthalmology for review, and therefore ocular injury data in the current study are based on subgroup analysis of only patients who were formally reviewed by an ophthalmologist. Baseline characteristics and mechanisms of injury were similar between those reviewed by ophthalmology, and those that did not have a formal review (**Appendix 1**). Prior research suggests that in the presence of a normal visual acuity, normal pupil responses, minimal gaze-evoked pain and full extraocular movements, few significant injuries are missed (9–11). A normal ophthalmic examination (normal VA and pupil responses) was documented in a large proportion of those not formally reviewed by ophthalmology in this study, suggesting an appropriate triaging process which is concordant with the screening criteria used in prior research (10, 11). Less than 1% of ocular injuries in our study required emergent ophthalmic surgery (*n* = 2), which indicates that the vast majority of ocular injuries occurring in the context of orbital fractures in our study population are able to be managed medically.

Our study was limited by nature of its retrospective design, and single-centre recruitment. Data availability within the electronic records reviewed was variable. Minimal age and ethnicity data were missing from the dataset reviewed and therefore demographic statistics were well captured by our study, however, inconsistent standards with regard to documentation of initial ophthalmic examination findings at presentation may have confounded results. We anticipate that some minor injuries (such as subconjunctival hemorrhage) may not have been captured in this study, however, moderate and major ocular injuries prompting ophthalmic review or intervention are well represented in our dataset. We did not gather information regarding delayed ophthalmic specialty input for patients who may have been initially reviewed by a non-ophthalmic medical practitioner, and therefore we are unable to formally comment on the number of missed ophthalmic injuries in our patient cohort.

Of the orbital fractures reviewed by ophthalmology in our study population, 33% had an associated ocular injury, with no significant difference in injury rates between isolated and complex fracture patterns. Complex fractures were less likely to have a documented ophthalmic review. Mechanisms of injury and demographics involved are consistent with existing published literature, with interpersonal violence the most common mechanism resulting in ocular injury across both complex and isolated fracture patterns. Current practice with regard to ophthalmology review in the acute setting appears to be safe, however, further research exploring rates of ocular injury in visually asymptomatic patients within this cohort is required.

## Data availability statement

The raw data supporting the conclusions of this article will be made available by the authors, without undue reservation.

## Ethics statement

The studies involving humans were approved by Te Whatu Ora Waitaha Canterbury Research and Ethics Committee. The studies were conducted in accordance with the local legislation and institutional requirements. Written informed consent for participation was not required from the participants or the participants’ legal guardians/next of kin in accordance with the national legislation and institutional requirements.

## Author contributions

NT: Data curation, Formal Analysis, Investigation, Methodology, Project administration, Writing – original draft, Writing – review & editing. PN: Data curation, Methodology, Project administration, Writing – review & editing. SN: Formal Analysis, Investigation, Methodology, Resources, Supervision, Writing – review & editing. RS: Methodology, Resources, Supervision, Writing – review & editing. JE: Supervision, Writing – review & editing.

## References

[1] Moore BK Smit R Colquhoun A Thompson WM. Maxillofacial fractures at Waikato Hospital, New Zealand: 2004 to 2013. N Z Med J (2015) 128(1426):96–102. 26913913

[2] Iftikhar M Canner JK Hall L Ahmad M Srikumaran D Woreta FA. Characteristics of orbital floor fractures in the United States from 2006 to 2017. Ophthalmology (2021) 128(3):463–70. doi: 10.1016/j.ophtha.2020.06.065 32659309

[3] Asiri M Aldowah O. Ocular findings in patients with orbital fractures: A 1-year prospective study in a tertiary center. Med Kaunas Lith (2023) 59(6):1091. doi: 10.3390/medicina59061091 PMC10305705 37374295

[4] Ho TQ Jupiter D Tsai JH Czerwinski M. The incidence of ocular injuries in isolated orbital fractures. Ann Plast Surg (2017) 78(1):59–61. doi: 10.1097/SAP.0000000000000748 26835822

[5] Rossin EJ Szypko C Giese I Hall N Gardiner MF Lorch A. Factors associated with increased risk of serious ocular injury in the setting of orbital fracture. JAMA Ophthalmol (2021) 139(1):77–83. doi: 10.1001/jamaophthalmol.2020.5108 33237267 PMC7689570

[6] Zhong E Chou TY Chaleff AJ Scofield-Kaplan SM Perzia BM Naqvi J. Orbital fractures and risk factors for ocular injury. Clin Ophthalmol (2022) 16:4153–61. doi: 10.2147/OPTH.S391175 PMC9760578 36544896

[7] Monk JHG Thomson WM Tong DC. Trends in maxillofacial fractures in Otago-Southland, New Zealand: 2009 to 2020. N Z Med J (2022) 135(1557):76–87. 35772115

[8] Anand L Sealey C. Orbital fractures treated in Auckland from 2010-2015: review of patient outcomes. N Z Med J (2017) 130(1458):21–6. 28694536

[9] Andrews BT Jackson AS Nazir N Hromas A Sokol JA Thurston TE. Orbit fractures: Identifying patient factors indicating high risk for ocular and periocular injury. Laryngoscope (2016) 126(S4). doi: 10.1002/lary.25805 26690301

[10] Chow J Parthasarathi K Mehanna P Whist E. Primary assessment of the patient with orbital fractures should include pupillary response and visual acuity changes to detect occult major ocular injuries. J Oral Maxillofac Surg (2018) 76(11):2370–5. doi: 10.1016/j.joms.2018.04.024 29782814

[11] Richani K Do TH Merritt HA Pfeiffer ML Chuang AZ Phillips ME. Screening criteria for detecting severe ocular injuries in the setting of orbital fractures. Ophthal Plast Reconstr Surg (2019) 35(6):609–14. doi: 10.1097/IOP.0000000000001422 PMC6834889 31162302

[12] Magarakis M Mundinger GS Kelamis JA Dorafshar AH Bojovic B Rodriguez ED. Ocular injury, visual impairment, and blindness associated with facial fractures: A systematic literature review. Plast Reconstr Surg (2012) 129(1):227–33. doi: 10.1097/PRS.0b013e3182362a6d 21915081

