# Supplementary material for: Orbital fractures and concurrent ocular injury in a New Zealand tertiary centre
Source: Front Ophthalmol (Lausanne). 2023 Nov 21;3:1305528. doi: 10.3389/fopht.2023.1305528 (PMC11182195; doi:10.3389/fopht.2023.1305528)
Supplement: Supplementary file 1 [file Table_1.docx]

Supplementary Material

# Supplementary Data

**Appendix 1** Demographic characteristics by presence of absence of ophthalmic review

|  | | **Ophthalmic Review** | | **No Ophthalmic Review** | | **Total** | | |  |
| --- | --- | --- | --- | --- | --- | --- | --- | --- | --- |
|  | | **n** | **%** | **n** | **%** | ***p*-value*** | **n** | **%** |  |
| *Total Patients* | | 118 | 42 | 166 | 58 |  | 284 | 100 |  |
| *Mean Age (years)* | | 38 |  | 44 |  | 0.02 | 42 |  |  |
| *Male Gender* | | 88 | 75 | 129 | 77 | 0.77 | 214 | 76 |  |
| *Ethnicity* | |  |  |  |  |  |  |  |  |
| NZ European | | 85 | 72 | 118 | 70 |  | 203 | 72 |  |
| Māori | | 16 | 14 | 25 | 15 |  | 41 | 14 |  |
| Pasifika | | 4 | 3 | 9 | 5 |  | 13 | 4 |  |
| Asian | | 7 | 6 | 4 | 2 |  | 11 | 4 |  |
| Other | | 6 | 5 | 9 | 5 |  | 17 | 6 |  |
| *Mechanism* | |  |  |  |  |  |  |  | |
| Interpersonal violence | | 46 | 39 | 45 | 27 |  | 91 | 32 | |
| Sporting injury | | 15 | 13 | 35 | 21 |  | 50 | 18 | |
| Fall | | 20 | 17 | 45 | 27 |  | 65 | 23 | |
| Motor vehicle accident | | 7 | 6 | 4 | 2 |  | 11 | 4 | |
| Bicycle | | 4 | 3 | 16 | 10 |  | 20 | 7 | |
| E-scooter | | 4 | 3 | 11 | 7 |  | 15 | 5 | |
|  | Other | 22 | 19 | 10 | 6 |  | 32 | 11 | |

*p-values calculated using two-tailed tests of statistical significance
